# Olopatadine vs. Rupatadine for Allergic Rhinitis: A Meta-Analysis of Randomized Controlled Trials

**DOI:** 10.7759/cureus.90306

**Published:** 2025-08-17

**Authors:** Abdur Rehman, Muhammad Arshad, Namra Asif, Muhammad Shuaib Khan, Farhat Malik, Mohammad Moaviz Aslam, Shahroz Ansar, Mahtab Zafar, Imran Khan, Shahzaib Maqbool, Abdulqadir J Nashwan

**Affiliations:** 1 Otolaryngology - Head and Neck Surgery, Rawalpindi Medical University, Rawalpindi, PAK; 2 Otolaryngology, Watim Medical and Dental College, Islamabad, PAK; 3 Otolaryngology, Rawalpindi Medical University, Rawalpindi, PAK; 4 Otolaryngology, Rahbar Medical and Dental College, Lahore, PAK; 5 Internal Medicine, NYC Health and Hospitals/Woodhull, New York City, USA; 6 Internal Medicine, HCA Midwest Health, Kansas City, USA; 7 Nursing and Midwifery Research, Hamad Medical Corporation, Doha, QAT

**Keywords:** allergic rhinitis, histamine h1 antagonists, nasal symptoms, olopatadine hydrochloride, oral antihistamine, rupatadine, total nasal symptom score (tnss)

## Abstract

This systematic review and meta-analysis aimed to compare the efficacy and safety of olopatadine and rupatadine in the treatment of allergic rhinitis (AR). By synthesizing data from randomized controlled trials (RCTs), this study aimed to evaluate symptom reduction, specifically in terms of the Total Nasal Symptom Score (TNSS), and assess the incidence of adverse effects between these two second-generation antihistamines.

A comprehensive literature search was conducted in PubMed, Google Scholar, Web of Science, Embase, and Cochrane databases from November 2024 to February 2025. Studies were selected based on the Preferred Reporting Items for Systematic Reviews and Meta-Analyses (PRISMA) guidelines. Clinical trials comparing olopatadine and rupatadine in AR patients were included, with a focus on TNSS reduction and adverse event incidence. The Jadad scale was used for quality assessment. Data were analyzed using the DerSimonian and Laird random effects model, with mean differences and risk ratios (RR) calculated. Heterogeneity was assessed using the chi-square test and I² statistic. A total of four RCTs involving 304 patients (153 in the olopatadine group, 151 in the rupatadine group) met the inclusion criteria. Olopatadine demonstrated superior TNSS reduction (mean difference: 2.51; 95% CI: 1.58 to 3.44; p<0.0001), with low heterogeneity (I²=0%). Adverse events were comparable between groups (RR=0.66; 95% CI: 0.29-1.50; P=0.33), with dry mouth, headache, and mild sedation being the most frequently reported. Olopatadine showed greater efficacy in reducing symptoms of allergic rhinitis compared to rupatadine, while both drugs had similar safety profiles. These findings suggest that olopatadine may be preferred for more symptom relief, though larger trials are needed for further validation.

## Introduction and background

Allergic rhinitis (AR) is a common, chronic inflammatory condition of the nasal mucosa, mediated by immunoglobulin E (IgE) in response to allergens, such as pollen, dust mites, animal dander, and mold [1]. AR affects a significant proportion of the global population, with prevalence estimates ranging between 25% and 40% [2]. The hallmark symptoms include nasal congestion, rhinorrhea, sneezing, nasal pruritus, and, in some cases, ocular symptoms, such as itching and redness [3]. These symptoms can significantly impair quality of life, productivity, and cognitive function [4].

Histamine plays a crucial role in the pathophysiology of AR by binding to H1 receptors, leading to vasodilation, increased vascular permeability, and nerve activation, which collectively contribute to the characteristic symptoms of the disease [5]. Antihistamines, particularly second-generation H1 receptor antagonists, serve as a cornerstone of pharmacological management due to their ability to counteract histamine’s effects while minimizing sedation and other central nervous system side effects associated with first-generation antihistamines [6].

Rupatadine is a second-generation antihistamine with dual-action properties, functioning as both an H1 receptor antagonist and a platelet-activating factor (PAF) receptor antagonist [2]. PAF plays a role in eosinophil recruitment and the exacerbation of the inflammatory response in AR, making rupatadine particularly effective in symptom control [7]. Studies have demonstrated rupatadine’s rapid onset of action and sustained efficacy in reducing nasal and ocular symptoms [5]. Additionally, a network meta-analysis found that rupatadine 20 mg ranked highest in terms of total symptom score reduction compared to other H1-antihistamines [5].

Olopatadine, another second-generation antihistamine, selectively blocks H1 receptors and exhibits additional anti-inflammatory effects by inhibiting the release of inflammatory mediators, such as leukotrienes and thromboxane, from eosinophils and mast cells [6]. Clinical trials suggest that olopatadine is effective in reducing nasal obstruction and controlling overall AR symptoms, possibly due to its dual mechanism of action [3]. Some studies have suggested that olopatadine may have a superior safety profile compared to rupatadine, with fewer reported adverse effects [4].

Despite the established efficacy of both olopatadine and rupatadine, comparative analyses regarding their relative benefits remain limited. Some studies suggest that rupatadine is more effective in alleviating nasal symptoms, while others favor olopatadine due to its superior safety profile [4,6]. Given these discrepancies, a systematic meta-analysis is warranted to provide a robust comparison of the two antihistamines.

AR is a prevalent condition characterized by commonly irritating symptoms. The Total Nasal Symptom Score (TNSS) is a widely used measure in AR research, combining these individual symptoms into a composite score to assess overall disease severity and treatment efficacy. While both olopatadine and rupatadine are second-generation antihistamines effective for AR, there is limited direct comparison of their efficacy and safety profiles. This meta-analysis addresses this evidence gap by synthesizing data from randomized controlled trials to provide a robust comparison of these two treatments, ultimately guiding clinical decision-making for optimal AR management.

This meta-analysis aimed to evaluate the relative efficacy of olopatadine and rupatadine in managing AR symptoms, as well as assess their safety profiles, including the incidence of adverse effects, and provide evidence-based recommendations for optimal AR management. By synthesizing data from multiple clinical studies, this analysis seeks to clarify the comparative effectiveness of olopatadine and rupatadine, ultimately aiding clinicians in selecting the most appropriate antihistamine for AR treatment.

## Review

Methods

This systematic review and meta-analysis followed a structured methodology to compare olopatadine and rupatadine for allergic rhinitis. We conducted a comprehensive literature search, selected randomized controlled trials based on predefined criteria, extracted relevant data, assessed study quality using the Jadad scale, and analyzed outcomes using a random effects model. These steps, detailed below, ensure a rigorous and reproducible approach to synthesizing evidence.

Search Strategy

A comprehensive literature search was conducted through various databases, including PubMed, Google Scholar, Web of Science, Embase, and the Cochrane database, to retrieve articles of interest. The literature search was conducted from an unspecified starting date to February 2025, utilizing various MeSH terms, including “Olopatadine”, “Rupatadine”, and “Allergic Rhinitis.” The formal search string for PubMed is as follows: ("Olopatadine Hydrochloride"[MeSH Terms] OR "Olopatadine"[Title/Abstract] OR "Patanol"[Title/Abstract]) AND ("Rupatadine"[MeSH Terms] OR "Rupatadine"[Title/Abstract]) AND ("Rhinitis, Allergic"[MeSH Terms] OR "Allergic Rhinitis"[Title/Abstract] OR "Seasonal Allergic Rhinitis"[Title/Abstract] OR "Perennial Allergic Rhinitis"[Title/Abstract]). All randomized controlled trials (RCTs) retrieved through these MeSH terms from various databases and the ClinicalTrials.gov website were included for both primary and secondary screening. Preferred Reporting Items for Systematic Reviews and Meta-Analyses (PRISMA) guidelines were used as a standard tool for conducting this meta-analysis [8].

Study Selection

All RCTs involving patients with allergic rhinitis that used olopatadine and rupatadine medications were included in our study. Studies with missing data, such as those related to medical history, outcome variables of interest (TNSS, adverse outcomes), demographics, and not fulfilling the standard criteria for allergic rhinitis, as well as studies with language other than English, were excluded from the final analysis of the meta-analysis. The process of primary and secondary screening was conducted by two authors (A.R. and S.M.), where each was blinded to the other. The assessment of study titles, abstracts, full-text articles, and relevant references was performed using standardized PRISMA guidelines. Any dispute in study selection was handled by a third author (M.S.K.). The population, intervention, control, and outcomes (PICO) criteria of the study are given in Table 1.

**Table 1:** PICO criteria applied to patients in this study taking olopatadine and rupatadine for allergic rhinitis. TNSS: Total Nasal Symptom Score; PICO: population, intervention, control, and outcomes

Variables	Characteristics
Population	Patients with allergic rhinitis
Intervention	Olopatadine
Comparator	Rupatadine
Outcome	Total Nasal Symptom Score (TNSS), composite adverse events

Data Extraction

The standard variables of interest, such as author name, year of study, country of study, gender-wise distribution, mean age of patients, type of treatment (olopatadine and rupatadine), study type, and duration, were extracted and presented in Table 2. Disease-specific variables, such as total nasal symptom score, composite adverse effects, type of drug administered, and follow-up duration, were extracted. The extraction was done by two separate authors, blinded to each other (M.A. and N.A.), and any differences were resolved by the third author (M.S.K.) to reduce bias.

**Table 2:** Study characteristics and demographics. TNSS: Total Nasal Symptom Score; QoL: quality of life; M: male; F: female; N/A: not available

Studies	Sample size (olopatadine/rupatadine)	Mean age (years) (M/F)	Gender distribution (M/F)	Study duration (weeks)	Primary outcome measures
Dnyandeo et al. (2020) [2]	50/50	35.9/34.3	60 M/40 F	4	TNSS, adverse effects
Dakhale et al. (2016) [4]	34/33	37.3/36.2	21 M/18 F	4	TNSS, adverse effects
Maiti et al. (2011) [6]	29/28	36.5/38.7	42.9% M/48.6% F	4	TNSS, QoL
Patel et al. (2024) [3]	40/40	N/A	22 M/17 F	4	TNSS, QoL (limited)

Quality Assessment

All randomized controlled trial studies included after secondary screening were assessed through a well-established “Jadad scale," which refers to a system used to assess the methodological quality of a randomized controlled trial (RCT) by assigning a score between 0 and five, where a score of three or higher is generally considered to indicate a high-quality study [9]. All trials with a score of three and above were included in the final synthesis of the data.

Data Analysis

The DerSimonian and Laird random effects model was used to pool risk ratios (RRs) and mean differences of TNSS, along with 95% confidence intervals (CIs), for the outcome variables. The chi-square test and I^2^ statistic were used to assess heterogeneity among studies. P<0.10 was considered statistically significant for the chi-square test. Heterogeneity was assessed using the I² statistic, with I² <25% considered low and values above 25% indicating moderate to high heterogeneity. This helped us in deciding which effect model to use for forest plot generation. If I2 was <25%, the fixed effect model was used; otherwise, the random effects model was applied. The analysis was conducted in Review Manager (RevMan) version 5.4 (Copenhagen, Denmark: The Cochrane Collaboration).

Results

Search Result

**Figure 1:**
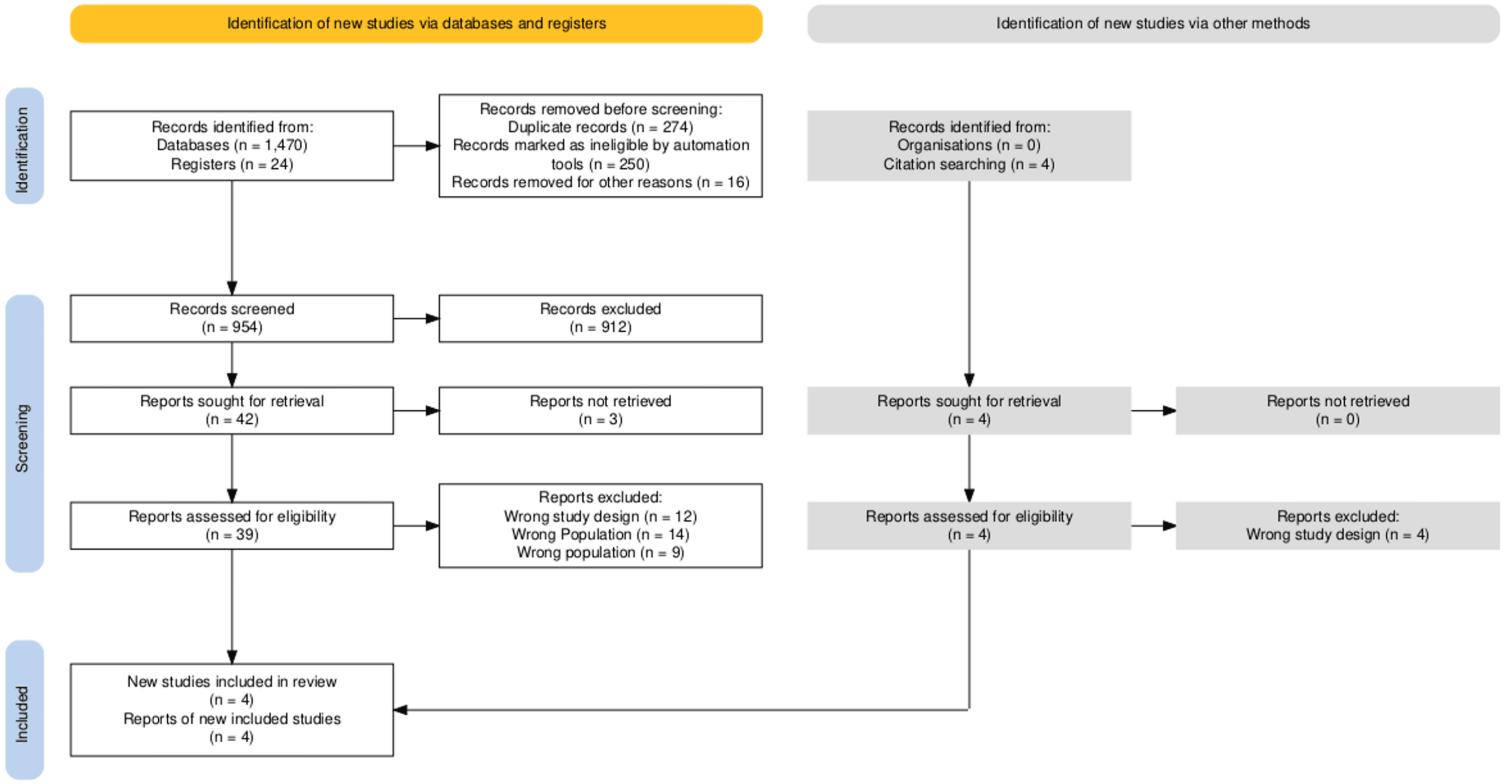
PRISMA flowchart for the study showing the search and screening process and the selection of the final eligible studies. PRISMA: Preferred Reporting Items for Systematic Reviews and Meta-Analyses

With an initial search, about 342 articles of interest were retrieved. After eliminating duplication and irrelevant studies (40), 10 studies were assessed for eligibility, and only four studies met our criteria for research question and quality. All of them were RCTs. The PRISMA flow chart for the selection of the final four studies is presented in Figure 1 [2-4,6].

Study Characteristics

A total of four RCTs were included in this meta-analysis, comparing the efficacy and safety of olopatadine and rupatadine in patients with AR. The studies, conducted between 2011 and 2024, included a total of 304 participants, with 153 in the olopatadine group and 151 in the rupatadine group. The mean age of participants ranged from 30.1 to 38.7 years, and 81 male participants and 72 female participants were included across the studies. The duration of each study was four weeks, allowing for a comparable assessment of treatment effects. The baseline characteristics of the included studies are summarized in Table 2. The primary outcomes assessed in the included trials were TNSS reduction and adverse effects.

Primary Outcome: TNSS

The primary outcome evaluated across all included studies was the TNSS, which measures the severity of symptoms associated with allergic rhinitis. The pooled analysis demonstrated a significant reduction in TNSS scores in both treatment groups; however, olopatadine showed a greater reduction in symptoms compared to rupatadine. The mean difference in TNSS reduction was 2.51 (95% CI: 1.58-3.44, p<0.0001), indicating a statistically significant advantage for olopatadine. The heterogeneity across the included studies was low (I²=0%), suggesting consistency in the observed treatment effect.

**Figure 2:**
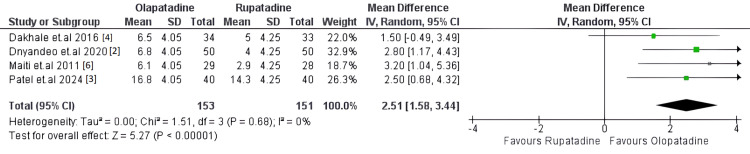
Forest plot of Total Nasal Symptom Score (TNSS) reduction. This plot compares TNSS reduction between olopatadine and rupatadine across four randomized controlled trials (RCTs). Olopatadine showed a significantly greater reduction in symptoms with low heterogeneity (I²=0%).

Individual study results largely supported the overall finding that olopatadine was more effective in reducing nasal symptoms than rupatadine. However, the study by Dakhale et al. showed overlapping confidence intervals, suggesting that the difference between the two treatments in that particular study was not as pronounced as in the others [4]. The forest plot illustrating TNSS reduction across the studies is presented in Figure 2 [2-4,6].

Secondary Outcome: Adverse Effects

Adverse effects were reported in all four included studies, with a higher incidence in the rupatadine group. The olopatadine group experienced between two and 10 adverse events per study, totaling 17 reported cases, while the rupatadine group experienced between four and 13 adverse events per study, totaling 25 cases. The pooled analysis revealed no statistically significant difference in adverse event occurrence between the two groups, with a risk ratio (RR) of 0.66 (95% CI: 0.29-1.50, p=0.33).

**Figure 3:**
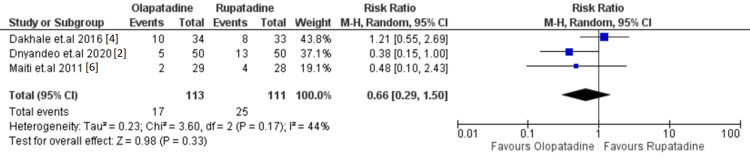
Forest plot of adverse events. This plot compares adverse event incidence between olopatadine and rupatadine. No significant difference was observed (p=0.33), with both drugs showing similar safety profiles.

Both treatments were well tolerated, with most reported adverse effects being mild and transient in nature. The most commonly observed side effects included dry mouth, headache, and mild sedation. These findings suggest that both drugs have a comparable safety profile. The forest plot for adverse events is presented in Figure 3 [2,4,6].

Quality of Life Outcomes

Data on quality of life outcomes were not consistently reported across all studies, making it difficult to conduct a pooled analysis. However, a narrative synthesis of the available results suggests that olopatadine may offer an advantage in improving quality of life, particularly in relation to sleep quality and nasal obstruction. The studies by Dnyandeo et al. and Maiti et al. reported improvements in these areas [2,6], while Patel et al. and Dakhale et al. did not provide QoL-specific data [3,4]. Furthermore, the use of validated QoL scales, such as the Rhinoconjunctivitis Quality of Life Questionnaire (RQLQ) or 36-Item Short Form Survey (SF-36), was inconsistent, making objective comparisons across studies challenging. These findings suggest a potential benefit of olopatadine in improving patient-reported outcomes, but more standardized assessments are needed to confirm this effect.

Risk of Bias Analysis

A risk of bias assessment was performed to evaluate the methodological quality of the included studies. Three studies demonstrated a low risk of bias, while Maiti et al. had an unclear risk in the domains of random sequence generation and allocation concealment [6]. The studies were generally well-conducted, with adequate blinding of participants and assessors, thereby minimizing the potential for performance bias. Attrition bias was low across all studies, as the loss to follow-up was minimal. Similarly, there was no evidence of selective reporting bias, as all studies reported the predefined outcomes outlined in their protocols. The summary of the risk of bias assessment is presented in Table 3 [9].

**Table 3:** Risk of bias summary using the Jadad scale. ROB: risk of bias

Studies	Random sequence generation	Allocation concealment	Blinding	Attrition bias	Reporting bias	Overall ROB rating
Dnyandeo et al. (2020) [2]	Low	Low	Low	Low	Low	Low
Dakhale et al. (2016) [4]	Low	Low	Low	Low	Low	Low
Maiti et al. (2011) [6]	Unclear	Unclear	Low	Low	Low	Moderate
Patel et al. (2024) [3]	Low	Low	Low	Low	Low	Low

Overall Interpretation

The findings of this meta-analysis indicate that olopatadine is more effective than rupatadine in reducing allergic rhinitis symptoms, as measured by a mean TNSS reduction of 2.51 (95% CI: 1.58-3.44, p<0.0001). This reduction is clinically meaningful, as prior research suggests that a TNSS decrease of approximately 2 points or greater is associated with noticeable improvements in patient-perceived symptom relief and daily functioning [10]. The superior efficacy of olopatadine was consistent across all included studies, with a statistically significant treatment effect and low heterogeneity (I²=0%). In terms of safety, both drugs demonstrated comparable tolerability, with no significant difference in adverse events (RR=0.66; 95% CI: 0.29-1.50, p=0.33). The available evidence also suggests that olopatadine may offer additional quality of life benefits, particularly in sleep quality and nasal obstruction; however, the lack of standardized QoL assessments across studies limits the strength of this conclusion.

Discussion

This meta-analysis comparing the efficacy and safety of olopatadine and rupatadine in the management of allergic rhinitis (AR) found that olopatadine was more effective in reducing the Total Nasal Symptom Score (TNSS) than rupatadine. The pooled data demonstrated a statistically significant reduction in TNSS for olopatadine, indicating superior symptom relief, particularly for nasal congestion and sneezing. These findings align with previous studies demonstrating olopatadine's dual mechanism of action as both an H1-antihistamine and an anti-inflammatory agent, which contributes to its effectiveness in treating allergic rhinitis [11]. More recent studies, such as those by Ridolo et al. and Klimek et al., further support olopatadine’s strong anti-inflammatory properties, reinforcing its superior efficacy in AR management [12,13].

In terms of adverse effects, both drugs were generally well tolerated. However, rupatadine was associated with a slightly higher incidence of mild sedation and dry mouth, consistent with its known pharmacological profile [4]. This finding aligns with more recent studies by Dnyandeo et al., who reported that olopatadine has a better safety profile, with fewer reports of sedation-related side effects [2]. No serious adverse events were reported in any of the included studies, indicating that both drugs are safe for clinical use.

The safety profiles of oral olopatadine and rupatadine have been well-documented in prior research. A study by Yamamoto et al. reported the most common adverse effects of oral olopatadine hydrochloride as somnolence (2.7%), headache (1.2%), dry mouth (0.8%), and fatigue (0.6%), all categorized as mild to moderate in severity [11]. Similarly, Picado identified the most frequent adverse effects of rupatadine as somnolence (9.5%), headache (6.8%), fatigue (3.2%), dry mouth (1.9%), and dizziness (1.7%), also mild to moderate [14]. No severe or life-threatening adverse effects were noted for either drug in these studies, consistent with the safety outcomes observed in our meta-analysis.

The findings of this study align with previous research on second-generation antihistamines. A network meta-analysis by Hong et al. found that rupatadine 20 mg was one of the most effective oral antihistamines for AR symptom relief. However, olopatadine was not included in their comparison [5]. However, other head-to-head RCTs have shown that olopatadine provides superior TNSS reduction and better improvement in quality of life compared to rupatadine [15,16]. More recent studies, such as those by Klimek et al., further validate these results, reinforcing the superior clinical efficacy of olopatadine [13].

The superior efficacy of olopatadine is attributed to its strong H1-receptor antagonism and additional anti-inflammatory effects, such as the inhibition of leukotrienes and thromboxane A2, which play a significant role in nasal inflammation [16]. On the other hand, rupatadine blocks both H1-receptors and platelet-activating factor (PAF), which theoretically provides broader anti-inflammatory effects. Still, this mechanism has not translated into greater clinical effectiveness in AR treatment [17]. Recent pharmacoeconomic analyses, such as those by Hossenbaccus et al. and Muñoz-Cano et al., suggest that despite rupatadine’s additional PAF-inhibitory effects, olopatadine remains the more effective choice in terms of symptom relief and cost-effectiveness [18,19].

Given the findings, olopatadine may be preferred for AR patients requiring stronger symptom relief, particularly for nasal congestion and sneezing. Rupatadine remains a viable alternative, particularly in patients who may benefit from its PAF-inhibitory effects, though its clinical relevance in AR is still debated [20]. More recent discussions, such as those by Lipiec and Jurkiewicz, continue to question the clinical advantage of PAF inhibition in AR treatment [21].

Both drugs have comparable safety profiles, making them suitable for long-term use. However, clinicians should individualize treatment choices based on patient preferences, tolerability, and cost-effectiveness. Some studies suggest that olopatadine may be more cost-efficient due to its higher efficacy, reducing the need for additional symptom management [4]. This notion is reinforced by recent cost-effectiveness analyses by Hossenbaccus et al. [18].

This study has several strengths that enhance the reliability of its findings. The inclusion of RCTs minimizes the risk of bias, ensuring a higher level of evidence. Additionally, the analysis demonstrated low heterogeneity (I²=0%), indicating consistent results across studies. The use of validated outcome measures, such as the TNSS and QoL assessments, further strengthens the study’s ability to accurately evaluate the efficacy of olopatadine and rupatadine in the management of allergic rhinitis [5].

However, certain limitations should be acknowledged. The small sample size (304 participants) restricts the generalizability of the findings to a broader population. Additionally, the lack of standardized QoL assessments across studies makes it difficult to compare improvements in patient well-being comprehensively. Potential publication bias is also a concern, as most of the included studies were conducted in Asia, which may limit the applicability of the results to other populations. Furthermore, unclear risk of bias in certain studies, such as Maiti et al., due to incomplete reporting of randomization, raises concerns regarding the methodological rigor of some trials [6]. Recent studies, such as those by Dnyandeo et al., further highlight the need for more standardized methodologies in AR trials [2].

To address these limitations, future research should focus on conducting larger, multicenter RCTs across diverse populations, incorporating standardized QoL assessments like the RQLQ to improve comparability. Additionally, head-to-head comparisons of olopatadine and rupatadine at different doses (e.g., rupatadine 20 mg) should be explored to assess potential dose-dependent efficacy variations. Long-term studies evaluating the safety and efficacy of both drugs in the management of chronic allergic rhinitis would further contribute to evidence-based treatment recommendations [12].

## Conclusions

This systematic review and meta-analysis demonstrate that both olopatadine and rupatadine are effective and generally well-tolerated options for treating allergic rhinitis. However, olopatadine may offer a slight advantage in terms of symptom relief based on pooled evidence from randomized controlled trials.

Given the observed greater reduction in TNSS, olopatadine could be a more suitable option for patients with moderate to severe nasal symptoms, though both agents remain valid first-line treatments. This finding highlights the importance of considering individual patient symptoms and preferences when selecting an antihistamine for allergic rhinitis.
